# Healthcare associated infections (HAI) in long-term care facilities in Europe

**DOI:** 10.1186/1753-6561-5-S6-P157

**Published:** 2011-06-29

**Authors:** ML Moro, B Jans, K Latour, E Ricchizzi, B Cookson, D MacKenzie, M Van de Mortel, J Fabry, HALT national networks

**Affiliations:** 1 ASSR, Bologna, Italy; 2 IPH, Brussels, Belgium; 3 HPA, London, UK; 4 UCB, Lyon, France

## Introduction / objectives

The HALT Project (HAI in European Long-Term Care Facilities) has been funded by the ECDC in 2009, to develop and implement a sustainable methodology to estimate the prevalence of infections and antimicrobial use, and to assess the status of infection control programmes in Europe. A pilot point prevalence survey (PPS) was conducted in November 2009 and an European wide PPS in May-September 2010.

## Methods

High skilled nursing homes (NHs) were included, on a voluntary basis. Each facility collected on a single day: 1) institutional data; 2) aggregated data on residents’ characteristics; 3) individual data for residents on systemic antibiotics and/or with an infection the day of the survey. Infections were diagnosed according to McGeer definitions, but the physician diagnosis was also recorded. An ad hoc software was developed for data input, providing a report to each facility. A questionnaire was completed by national representatives.

## Results

A total of 13 countries, 117 NHs and 14,491 residents participated to the pilot PPS. The survey was perceived as feasible (median 5.6 hours/100 beds for data collection, range 1.1-40) and easy. Characteristics of the facility, care load indicators, and risk factors varied widely among facilities and countries as well as antimicrobial stewardship activities and availability of infection control resources. In May-September 2010, 28 countries and 722 NHs have joined the European PPS accounting for more than 60,000 residents.

## Conclusion

Resources available in NHs for surveillance are limited. The HALT methodology may be feasible to assess infection and antibiotic use in this setting.

## Disclosure of interest

None declared.

